# A protein chimera strategy supports production of a model “difficult‐to‐express” recombinant target

**DOI:** 10.1002/1873-3468.13170

**Published:** 2018-07-03

**Authors:** Hirra Hussain, David I. Fisher, Robert G. Roth, W. Mark Abbott, Manuel Alejandro Carballo‐Amador, Jim Warwicker, Alan J. Dickson

**Affiliations:** ^1^ Faculty of Science and Engineering Manchester Institute of Biotechnology University of Manchester UK; ^2^ Discovery Biology, Discovery Sciences IMED Biotech Unit AstraZeneca Cambridge UK; ^3^ Discovery Biology, Discovery Sciences IMED Biotech Unit AstraZeneca Gothenburg Sweden; ^4^ Present address: Peak Proteins Alderley Edge Cheshire SK10 4TG UK; ^5^ Present address: Facultad de Ciencias Universidad Autónoma de Baja California Km. 103 Carretera Tijuana – Ensenada, Pedregal Playitas Ensenada 22860 Baja California Mexico

**Keywords:** difficult‐to‐express, mammalian expression system, predictive computational tool, protein engineering, recombinant protein production, tissue inhibitor of metalloproteinase

## Abstract

Due in part to the needs of the biopharmaceutical industry, there has been an increased drive to generate high quality recombinant proteins in large amounts. However, achieving high yields can be a challenge as the novelty and increased complexity of new targets often makes them ‘difficult‐to‐express’. This study aimed to define the molecular features that restrict the production of a model ‘difficult‐to‐express’ recombinant protein, Tissue Inhibitor Metalloproteinase‐3 (TIMP‐3). Building from experimental data, computational approaches were used to rationalize the redesign of this recombinant target to generate a chimera with enhanced secretion. The results highlight the importance of early identification of unfavourable sequence attributes, enabling the generation of engineered protein forms that bypass ‘secretory’ bottlenecks and result in efficient recombinant protein production.

## Abbreviations

**ARTN**, Artemin

**CDR3**, complementary determining region 3

**EndoH**, Endoglycosidase H

**LRP1**, low‐density lipoprotein receptor‐related protein 1

**PAI‐1**, murine plasminogen activator inhibitor

**PDB**, Protein Data Bank

**PNGase F**, N‐Glycosidase F

**TIMPs**, Tissue inhibitors of metalloproteinases

Over recent years the use of mammalian expression systems has increased for the production of approved biotherapeutics 1, 2. Efficient recombinant protein production of secreted targets in mammalian cells requires the balance between different steps/processes as proteins process through the complex secretory pathway 3, 4, 5. Limitations in the secretion of recombinant proteins can impact both protein quality and yield, which can have a negative impact on downstream processes. Published reports have focused on the characterization of such ‘difficult‐to‐express’ recombinant proteins and described the modification of culture conditions 6, 7, 8, 9, 10, 11, 12, 13, 14, 15 or the design of appropriate cell/protein engineering strategies to overcome restrictions in their production 15, 16, 17, 18, 19, 20, 21, 22, 23, 24, 25, 26. However, little is known regarding the mechanisms underpinning poor recombinant protein production, particularly between proteins of high sequence similarity.

The amino acid sequence (primary structure) has effects on protein folding, modification, stability and solubility 27, 28, 29. Reports surrounding production of monoclonal antibodies (mAbs) suggest that unique sequence features influence the effectiveness of recombinant protein production 30, 31, 32. For bacterial expression systems, computational tools have been implemented to predict of surface properties to allow rational design of recombinant targets with increased protein solubility of otherwise insoluble or poorly soluble target proteins 29, 33, 34, 35, 36. Previous work has established that charge and polarity influences protein solubility 37. This study has applied a predictive computational approach to the context of mammalian recombinant protein production.

Tissue inhibitors of metalloproteinases (TIMPs) regulate the activity of matrix metalloproteinases 38, 39, 40, 41. The three‐dimensional structure of TIMPs consists of two domains, a larger N‐terminal and smaller C‐terminal domain, each stabilized by conserved intra‐ and interdomain disulfide bonds 42, 43, 44, 45. Previous work by our group with members of the TIMP family (TIMP‐2, TIMP‐3 and TIMP‐4) show differences in their expression (secretion) in mammalian cells despite high sequence and structural similarity 26.

In this study, the difference in secretion of proteins of close sequence similarity was examined by analysis of the consequences of amino acid sequence features and protein structure. Analyses focused on two model proteins, TIMP‐2 and ‘difficult‐to‐express’ TIMP‐3, that have very significant sequence identity/similarity but grossly different expression in mammalian cell expression systems 26. The characterization of protein production, and identification of limiting steps in TIMP‐3 production was the subject of an earlier study 26. Here, we describe the application of a computational tool 36, 37, 46 coupled to protein engineering strategies, to understand the influence of amino acid sequence on TIMP‐3 production. Computational screening identified amino acid sequence features that contributed to poor TIMP‐3 secretion and these data rationalized a redesign of a TIMP‐3 chimera that overcame the block in secretion of this ‘difficult’ target. Furthermore, computational screening of other secreted target proteins, of different sequence and structure, identified unfavourable sequence/structural features that predicted expressability offering avenues for enhanced secretion.

## Materials and methods

### Materials

All materials were sourced of the highest purity from Sigma‐Aldrich unless stated otherwise.

### Constructs

**Figure 1:**
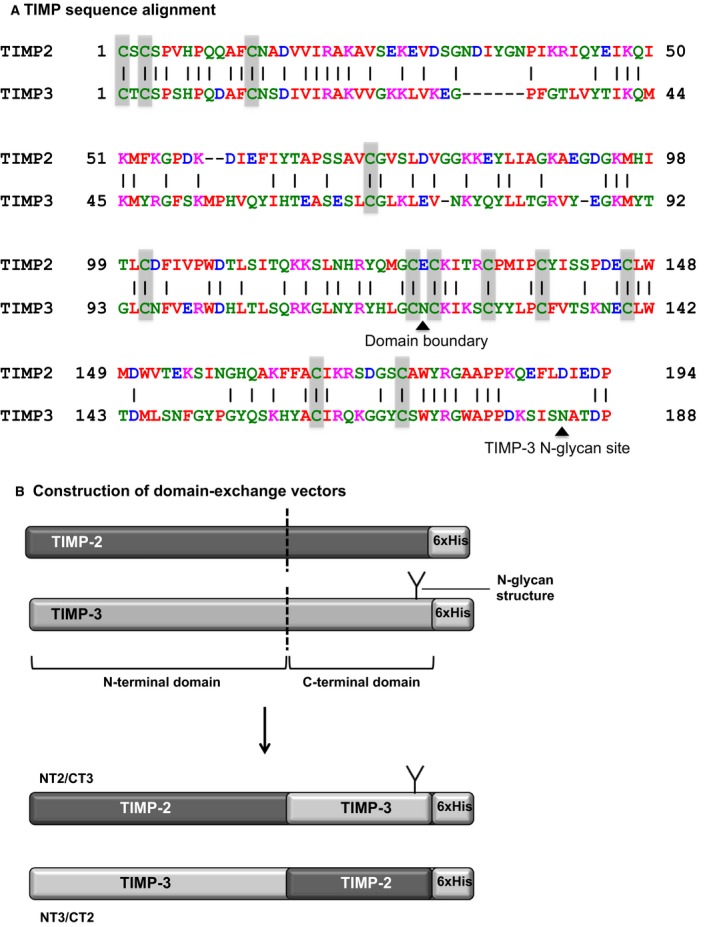
Alignment of TIMP‐2 and TIMP‐3 amino acid sequences. (A) Amino acid sequences for TIMP‐2 and TIMP‐3 were chemically annotated and aligned using EMBOSS Needle tools 59, 60. Amino acids are coloured according to their respective properties, Small/Hydrophobic (red), acidic (blue), basic (magenta), hydroxyl/sulfhydryl or amine (green). Conserved amino acids (I) and gaps in the alignment (−) are shown. Conserved cysteine residues for disulphide bond formation (grey boxes), the boundary between the N‐terminal and C‐terminal TIMP domains and the TIMP‐3 N‐glycosylation site are also indicated. (B) Schematic diagram summarising the construction of domain‐exchanged vectors is shown.

All gene inserts were synthesized, codon‐optimized for mammalian expression systems and cloned into pDEST12.2*‐OriP* 26, 47. Rat genes for TIMP‐2, TIMP‐3 and TIMP‐4 were as described in Hussain *et al*. (2017). Fusion constructs were generated for TIMP‐2 and TIMP‐3 by exchanging gene sequences between the N‐ and C‐terminal domain boundary (amino acid positions 127 and 121 for TIMP‐2 and TIMP‐3, respectively, Fig. 1). An engineered form/chimera of TIMP‐3 (enTIMP‐3) was generated where amino acids K26‐I41 within the N‐terminal region of TIMP‐3 were replaced with the corresponding TIMP‐2 sequence (E26‐I47). All TIMP gene sequences carry an N‐terminal CD33 signal peptide and C‐terminal 6 × His tag. Human artemin (ARTN, 528 bp) and murine plasminogen activator inhibitor (PAI‐1, 1269 bp) both carry a native signal peptide and a N‐terminal 6 × His tag and 6 × HN (6 alternate histidine (H) and asparagine (N) residues) tag, respectively. All DNA solutions (1 mg·mL^−1^) were prepared in TE buffer (10 mm Tris, pH 8.0, 1 mm EDTA) under sterile conditions.

### Protein Expression in Chinese Hamster Ovary (CHO) cells

DNA constructs were expressed in a transient CHO expression system as described previously by Hussain *et al*. (2017) 26. Expression studies were scaled down *pro rata* in a total volume of 40 mL in 125 mL vented flasks (Corning^®^). Cell density and viability of transfected cultures was monitored daily using the trypan blue exclusion method. Cell pellets and culture supernatants were harvested by centrifugation (1000 × ***g***, 5 min) at specific time points (days 3, 5 and 6 post‐transfection) as described in Hussain *et al*. (2017) to determine protein expression patterns 26.

### Small‐scale protein purification

Proteins were purified using the 6 × His tag from culture supernatant samples using HIS‐Select^®^ nickel affinity gel (Sigma‐Aldrich, Dorset, UK) as per the manufacturer's instructions. All steps were performed at 4 °C. The purified sample (50 μL) was mixed with an equal volume of 2 × sample buffer (20% (v/v) glycerol, 125 mm Tris‐HCl, 4% (w/v) SDS, 0.01% (v/v) bromophenol blue) for SDS/PAGE and western blot analysis. Nonpurified (crude) samples were used as a comparison.

### SDS/PAGE

Cell culture medium and intracellular protein samples were resolved by SDS/PAGE as described previously 26. For reducing conditions, 1.8% (v/v) β‐mercaptoethanol (Sigma‐Aldrich) was added and the sample heated to 100 °C for 5 min to reduce and denature the proteins. Gels were stained with InstantBlue™ coomassie gel stain (Expedeon Inc.) and incubated with shaking for 15 min and destained with ddH_2_O. Gels were imaged using a Bio‐Rad Gel Doc system.

### Western blotting

Proteins separated by SDS/PAGE were transferred onto nitrocellulose membrane using a TE 22 wet transfer system (Thermo Fisher Scientific) according to the manufacturer's instructions.

Membranes were blocked in 5% (w/v) nonfat milk with 0.1% (v/v) Tween‐20 in phosphate buffered saline buffer (137 mm NaCl, 2.7 mm KCl, 10 mm Na_2_HPO_4_, 2 mm KH_2_PO_4_, pH 7.4, 5% mPBST) for 1 h at room temperature before incubation with primary antibodies in 5% mPBST for 1 h. Primary antibodies used in this study include an anti‐TIMP2 antibody (1 : 1000, cat no. ab53730, Abcam, Cambridge, UK), anti‐TIMP3 antibody (1 : 1000, cat no. ab39184, Abcam), anti‐6 × His tag (1 : 2000, cat no. ab18184, Abcam) and anti‐6 × HN tag (1 : 1000, cat no. 631213, Clontech Laboratories, California, USA). Extracellular‐signal‐regulated kinase 2 (anti‐ERK2 antibody, 1 : 1000, cat no. sc‐81459, Santa Cruz Biotechnology, Dallas, TX, USA) was used as a loading control for cell extracts. Following primary antibody incubation, blots were incubated with the appropriate IRDye^®^ 800CW secondary antibody (1 : 15000, LI‐COR Biosciences) in 5% mPBST. A peroxidase‐conjugated anti‐rabbit antibody (1 : 5000, cat no. 31460, Thermo Fisher Scientific, Waltham, MA, USA) was used for anti‐TIMP3 antibody detection. Proteins were detected either using infrared fluorescent imaging *via* a LI‐COR Odyssey^®^ Classic imager or using Pierce^®^ enhanced chemiluminescence western blotting substrate according to the manufacturer's instructions. Quantification of fluorescent western blots was completed using the LI‐COR Image Studio™ Lite software. All graphs were plotted and statistical analysis was performed in GraphPad Prism^®^ (Version 6.02).

### Glycosidase treatment

Culture medium and intracellular protein samples from day 5‐post transfection were treated with N‐Glycosidase F (PNGase F, Roche) and Endoglycosidase H (Endo H, New England Biolabs^®^) as described previously 26. Untreated and treated protein samples were subsequently analysed by western blot.

### Computational analysis

Structural models were generated for recombinant targets based on published structures from the Protein Data Bank (PDB) 48. Predicted structural models of TIMP‐2, TIMP‐3, TIMP‐4 and TIMP fusion/mutant sequences used in this study were generated using SWISS‐MODEL 49, 50, where the published structure of human TIMP‐2 (accession code: 1BR9) was used as a template. Published structures were also analysed for ARTN (accession code: 2GYZ) and PAI‐1 (accession code: 3LW2).

Sequence and structural predictions of protein solubility were obtained from computational work based on comparison with the solubility database of all *E. coli* proteins (eSOL) which contains the solubility distribution of 3173 *E. coli* proteins produced in a cell‐free expression system 51. It was found that the experimental solubility values (eSOL) were, on average, inversely correlated with size of calculated largest positive electrostatic potential patch 37. These calculations were made with the Finite Difference Poisson‐Boltzmann method, at pH 7 and ionic strength 0.15 Molar. Contouring of positive electrostatic potential was performed at the 25 mV level, and a threshold size derived that best separated the higher and lower solubility subsets of *E. coli* proteins 37. Values referred to as PosQ in this work report the ratio of maximum positive potential patch size to that threshold, so that higher PosQ values relate to larger maximal positive patch. A separate measure of the protein surface is the maximal ratio of nonpolar to polar solvent accessible surface area, over a given patch size. In this case, the patches are not contoured (as for electrostatic potential), but are generated from all atoms within 13 Å of a given central atom. This maximal value therefore gives an estimate of the degree of nonpolarity concentrated in a protein surface region, and may therefore relate to interactions with other molecules that are driven by nonpolar interactions. This measure has been used in previous work studying protein solubility 37, 46.

Following processing in the algorithm, visualization and analysis of structures was completed using the PyMOL™ Molecular Graphics System 52. The surface calculations produce coordinate files updated with either electrostatic potential or nonpolar to polar surface ratios in the B‐factor field, for convenient colour‐coding and visualization.

## Results

### Sequences within the N‐terminal domain limit TIMP‐3 production

We have shown that TIMP‐2 and TIMP‐3 were secreted to significantly different extents in a transient CHO expression system 26. Alignment of TIMP‐2 and TIMP‐3 amino acid sequences revealed discrete regions of extensive homology (44% identity and 67% similarity) but specific region(s) of significant amino acid sequence difference could not be defined (Fig. 1A). As a result, a protein engineering strategy was employed to identify regions of sequence that may affect protein production. Initial approaches exchanged conserved structural domains between TIMP‐2 and TIMP‐3 (Fig. 1B). Sequences for TIMP‐2 and TIMP‐3 were divided at the boundary of the larger N‐terminal and smaller C‐terminal domain and between conserved disulphide bonds (E127 and N121 for TIMP‐2 and TIMP‐3, respectively) (Fig. 1). Domain exchange resulted in two new DNA vectors. Fusion 1 (NT2/CT3) was made up of the N‐terminal domain of TIMP‐2 and C‐terminal domain of TIMP‐3 (that included the single N‐glycan site). Fusion 2 (NT3/CT2) contained the N‐terminal domain of TIMP‐3 and C‐terminal domain of TIMP‐2. Expression from constructs was screened transiently in the CHO‐EBNA‐GS cell line and protein assessed from cell extracts (intracellular) and culture medium (secreted) throughout the culture period.

**Figure 2:**
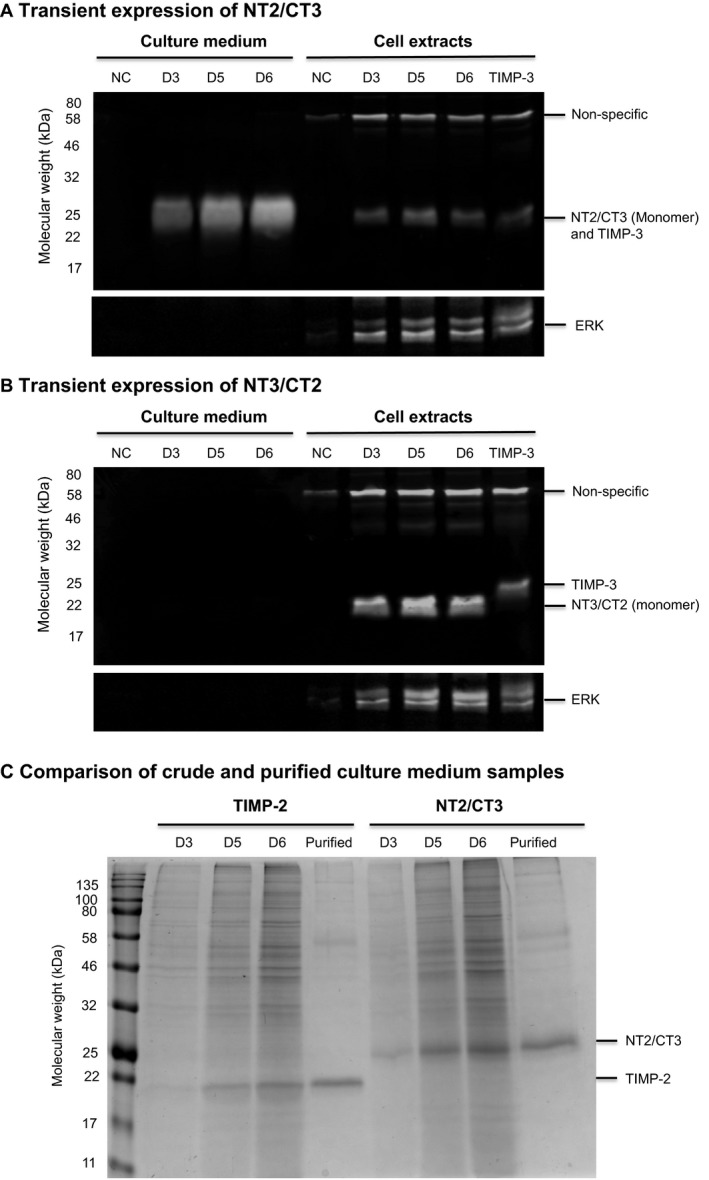
Transient expression of TIMP‐2 and TIMP‐3 domain‐exchanged sequences in CHO cell cultures. CHO‐EBNA‐GS cells were transiently transfected and sampled for secreted (culture medium) and intracellular (cell extracts) proteins on day 3, day 5 and day 6 (D3, D5 and D6) post‐transfection. Protein samples for, (A) NT2/CT3 and (B) NT3/CT2, were analysed by western blot. Nontransfected cells (NC) and intracellular TIMP‐3 (sampled day 5 post‐transfection) were loaded as controls. ERK was used as a loading control for cell extracts. The amount of TIMP‐2 and NT2/CT2 protein was assessed in crude and purified culture medium samples (sampled day 6 post‐transfection) by reducing SDS/PAGE (C). Data is representative of three biological replicates.

Western blot analysis using an anti‐6 × His detection antibody showed NT2/CT3 was detectable in increasing amounts in the culture medium from day 3 to day 6 (Fig. 2A). Intracellular NT2/CT3 protein was detected with a molecular weight similar to that of TIMP‐3, as expected. In contrast, NT3/CT2 was not detectable in the culture medium but was present inside cells at a molecular weight less than that of TIMP‐3, due to the absence of the N‐glycan site and glycan addition (Fig. 2B). These relative differences in protein expression were confirmed using specific antibodies to both TIMP‐2 and TIMP‐3 (Fig. S1).

The NT2/CT3 species detected in the culture medium appeared as a diffuse band, proposed to arise from glycan heterogeneity. Both NT2/CT3 intracellular and secreted protein were sensitive to PNGase F cleavage, confirming that proteins had been N‐glycosylated (Fig. S2a). Intracellular NT2/CT3 species was mainly Endo H‐sensitive, whereas a large proportion of secreted NT2/CT3 protein was Endo H‐resistant (Fig. S2a). These data suggest that the intracellular protein mainly existed in an immature high mannose N‐glycosylated form, whereas a mature form of the protein was secreted with processed glycan structures. Glycosidase treatment of intracellular NT3/CT2 protein confirmed it was not N‐glycosylated (data not shown). The data indicated that NT2/CT3 was successfully secreted, whilst NT3/CT2 was unable to complete post‐translational processing and was not secreted.

The addition of the C‐terminal portion of TIMP‐3 did not have a negative impact on the secretion of the TIMP‐2 N‐terminus (Fig. 2C). The results suggested that N‐terminal TIMP‐3 sequences were responsible for limitations in secretion. As the N‐termini of TIMP‐2 and TIMP‐3 contain multiple regions of amino acid difference (Fig. 1A), computational tools were employed for protein structure analysis to define specific regions within the TIMP‐3 N‐terminal domain that may act to restrict protein production.

### Computational analysis of TIMP protein structures reveals significant differences in surface properties

**Figure 3:**
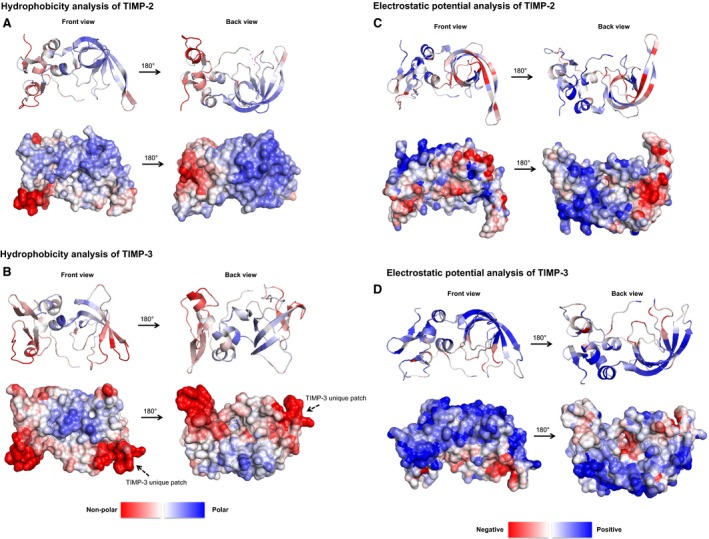
Hydrophobicity analysis of TIMP‐2 and TIMP‐3 protein structures. Structural models of TIMP‐2 and TIMP‐3 created using SWISS‐MODEL 49, 50 and processed through an algorithm developed by the Warwicker group (The University of Manchester). Predictions of the (A–B) hydrophobicity (nonpolar vs. polar regions) and (C–D) electrostatic potential (positive vs. negative charge) of protein surfaces were generated for TIMP‐2 (A,C) and TIMP‐3 (B,D). Proteins structures are represented as both tertiary structure ribbon representation (*top*) and surface maps (*bottom*) with a structural view of the front and back (180° rotation). Arrows indicate nonpolar patches unique to TIMP‐3. For electrostatic potential analysis, the predicted ranking of maximum positive electrostatic potential patch size (PosQ value) for TIMP‐2 and TIMP‐3 is detailed in Table 1.

Using the published structure of human TIMP‐2 (accession code: 1BR9) as a template, predicted structural models were produced using SWISS‐MODEL 49, 50 for TIMP‐2, TIMP‐3, NT2/CT3 and NT3/CT2 fusions (sequence of rat origin). Structures were analysed using reported methods 36, 37, 46 and predictions of protein surface hydrophobicity (Fig. 3A–B) and electrostatic potential (Fig. 3C–D) were obtained.

Hydrophobicity analyses categorized the surfaces into nonpolar (red) and polar (blue) regions. The majority of TIMP‐2 appeared weakly polar with two nonpolar patches located in the C‐terminal domain (Fig. 3A) which were also observed in TIMP‐3 (Fig. 3B). Two additional nonpolar patches unique to TIMP‐3 were observed in the N‐terminal domain. Annotation of amino acids located within these unique regions (between K26‐I41 and V56‐C68, Fig. 1A) revealed a high concentration of hydrophobic amino acids adjacent to basic lysines (K26, K27 and K30) that mainly locate to flexible loop regions. Comparing the native TIMP structures to the domain‐exchanged structures showed that NT2/CT3 resembled TIMP‐2 and NT3/CT2 was similar to TIMP‐3 (Fig. S3a). TIMP‐3 unique regions were also present in NT3/CT2. It is possible that exposed hydrophobic regions may act to abrogate efficient post‐translational processing and/or secretion of TIMP‐3 resulting in the lack of detectable TIMP‐3 and NT3/CT2 in the extracellular medium.

In parallel, the electrostatic potential of protein surfaces was examined. The electrostatic potential categorized sequence features into positively charged patches (blue) and negatively charged patches (red) associated with poor and good protein solubility in *E. coli,* respectively 36, 37, 46. TIMP‐2 surfaces showed the presence of small positively and negatively charged patches interspersed over the whole surface (Fig. 3C). In contrast, TIMP‐3 showed a large positively charged patch covering the top half of the structure with relatively few negatively charged patches visible (Fig. 3D). The ratio of maximum positive potential patch size to the threshold was reported as a numerical value (PosQ) summarized in Table 1. The PosQ value for TIMP‐3 (PosQ = 3.232) was higher than TIMP‐2 (PosQ = 1.768), which correlated with a larger maximal positive patch. The significant differences in the electrostatic potential reflect the difference in isoelectric points (pI) of TIMP‐2 (pI = 6.5) and TIMP‐3 (pI = 9.2) 40. As with the hydrophobicity analysis, surface maps of the domain‐exchanged/fusions showed that the electrostatic potential of NT2/CT3 resembled TIMP‐2 and NT3/CT2 was similar to TIMP‐3 (Fig. S3b). The large positive electrostatic potential patches seen for TIMP‐3 and NT3/CT2 were spread over multiple structure elements as well as exposed loop regions and did not localize to specific regions in the N‐terminus. Exchanging the domains between TIMP‐2 and TIMP‐3 increased the PosQ value for both NT2/CT3 (PosQ = 2.188) and NT3/CT2 (PosQ = 3.395).

**Table 1:** Summary of the maximum positive electrostatic potential patch size (PosQ value) obtained for all protein structures. The predicted experimental solubility relative to a solubility database of all *E. coli* proteins (eSOL) has shown to be inversely correlated with size of calculated largest positive electrostatic potential patch 37, 51. The PosQ value reports on the ratio of maximum positive potential patch size to that threshold, where a higher PosQ values relates to a larger maximal positive patch. This Table lists the protein structures in terms of increasing PosQ value and observed protein production profile in CHO cells

Structure	Predicted ranking (PosQ Value)	Observed protein secretion in CHO cells
mPAI‐1	1.132	Secreted
ARTN	1.539	Not secreted
TIMP‐2	1.768	Secreted
NT2/CT3	2.188	Secreted
enTIMP‐3	2.992	Secreted
TIMP‐4	3.026	Poorly secreted
TIMP‐3	3.232	Not secreted
NT3/CT2	3.395	Not secreted

Annotation of amino acids located inside the large positively charged patch of TIMP‐3 and NT3/CT2 revealed a higher proportion of basic amino acids (mainly lysine) in comparison to TIMP‐2 and NT2/CT3. In addition, some overlap was observed between the N‐terminal positively charged region and the previously identified TIMP‐3 and NT3/CT2 unique hydrophobic regions (Fig. 3B). The significance of basic amino acids identified in this study were consistent with published studies examining TIMP‐3 sequence features 53. Lee *et al*. (2007) identified basic amino acids unique to TIMP‐3 that resulted in an overall positively charged surface and responsible for the association of TIMP‐3 with the extracellular matrix. The study went on to mutate a number of these basic amino acids to generate a more soluble TIMP‐3 form, which had decreased affinity for the extracellular matrix and increasingly accumulated in the extracellular space 53. Observations by our group had shown TIMP‐3 was not sequestered to the extracellular matrix and did not interact with certain CHO host cell matrix proteins on the cell surface (data not shown). Of four basic amino acids (K26, K27, K30 and K76) identified in the N‐terminus by Lee *et al*. (2007), three basic amino acids (K26, K27 and K30) were also highlighted as significant in this current study.

### Protein engineering improves TIMP‐3 surface properties and results in successful production

**Figure 4:**
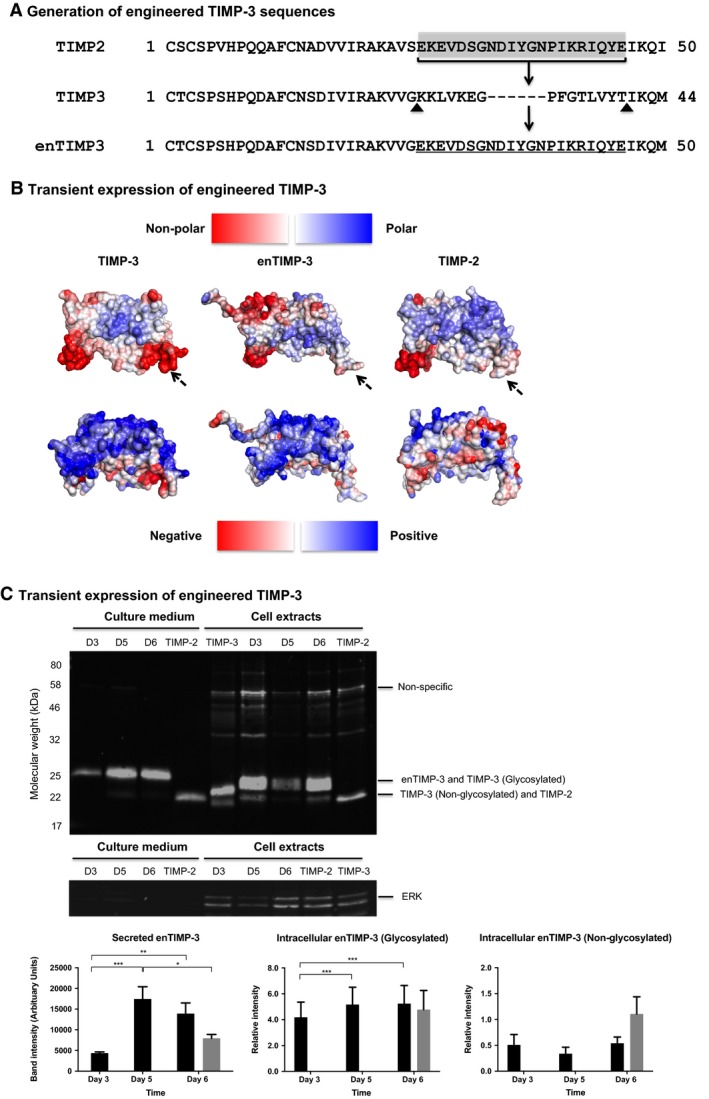
Computational analysis of an engineered TIMP‐3 chimera sequence (enTIMP‐3) and characterization of secreted and intracellular protein in CHO cell cultures. (A) Sequences within the N‐terminus of TIMP‐3 (positions 26‐41) were replaced with the corresponding TIMP‐2 sequence (positions 26‐47) to generate enTIMP‐3. (B) Predicted surface hydrophobicity (*top panel*) and electrostatic potential (*bottom panel*) are shown for TIMP‐3, enTIMP‐3 and TIMP‐2. The front view is shown for each structure and arrows indicate patches of interest. enTIMP‐3‐transfected CHO‐EBNA‐GS cultures were sampled for secreted and intracellular proteins on different days (D) throughout the culture period. (C) Protein samples were analysed by western blot with secreted TIMP‐2 and intracellular TIMP‐2 and TIMP‐3 loaded as controls (sampled day 6 post‐transfection). ERK was used as a loading control for intracellular samples. Western blots were quantified using the LI‐COR imaging system and plotted for secreted enTIMP‐3, glycosylated and nonglycosylated intracellular enTIMP‐3 forms, the corresponding controls (secreted TIMP‐2, glycosylated and nonglycosylated intracellular TIMP‐3) are also shown (*grey bars*). The data was analysed by two way ANOVA, where a *P* value of < 0.05 (*), < 0.01(**) and < 0.001(***) was deemed significantly different. Error bars shown are the mean value ± SEM of three biological replicates.

Using knowledge gained from protein engineering studies, computational analyses and published literature, a TIMP‐3 mutant/chimera was designed to evaluate its potential to decrease/eliminate problematic sequence features. A chimera was generated whereby a small region within the N‐terminus of TIMP‐3 was replaced with the corresponding sequence in TIMP‐2 (engineered TIMP‐3, enTIMP‐3, Fig. 4A). The TIMP‐3 chimeric sequence was generated with the aim of decreasing the electrostatic properties and hydrophobicity of the N‐terminus (Fig. 4B). The replaced region within TIMP‐3 was identified as being part of the large unique nonpolar patch and overlapped with regions identified in the electrostatic potential analyses. Replacement of this region also removed two of the three lysines unique to TIMP‐3 and added six extra amino acids derived from the TIMP‐2 sequence. enTIMP‐3 was processed through the algorithm to generate surface maps of electrostatic potential and hydrophobicity (Fig. 4B).

enTIMP‐3 displayed a significant decrease in the large hydrophobic patch, resembling TIMP‐2 structures (Fig. 4B*, top panel)*. Furthermore, enTIMP‐3 has a decreased PosQ value (2.992) compared to TIMP‐3 (Table 1). To empirically test whether this sequence modification resulted in successful secretion as predicted, enTIMP‐3 was cloned into pDEST12.2‐oriP and transiently expressed in CHO‐EBNA‐GS cells (Fig. 4C).

Intracellular and secreted protein was examined by western blot of enTIMP‐3 transfected cultures (Fig. 4C). enTIMP‐3 was detected in increasing amounts in the culture medium and cell extracts from day 3 to day 6. In addition, secreted enTIMP‐3 on day 5 was statistically significant from the secreted TIMP‐2 control (*P* value = 0.0311). The relative amount of intracellular glycosylated enTIMP‐3 species significantly increased between days 3 and 5 (*P* value = 0.0003) and day 3 and 6 (*P* value = 0.0002) and was comparable to the TIMP‐3 control. No significant difference in the relative amount of nonglycosylated enTIMP‐3 was observed with the TIMP‐3 control. As noted for NT2/CT3, the secreted and intracellular enTIMP‐3 species presented as diffuse bands. Glycosidase treatment of both intracellular and secreted enTIMP‐3 protein, suggested the intracellular enTIMP‐3 protein was of an immature N‐glycosylated form, whereas a fully processed mature form was secreted (Fig. S2b). Together, these data suggest the predictive computational approach, directing removal of N‐terminal nonpolar sequences and TIMP‐3 unique basic amino acids, resulted in the secretion of this modified, fully glycosylated form of TIMP‐3.

### Computational screening of other ‘difficult‐to‐express’ target proteins identifies potentially limiting sequence features

Computational analysis was extended to other target proteins to ascertain if the approach described may have a general applicability in identifying unfavourable sequence attributes. The extended panel of target proteins included Artemin (ARTN) and murine Plasminogen activator inhibitor 1 (PAI‐1) with different sequence and structural properties, as well as TIMP‐4 with high sequence similarity to TIMP‐2 and TIMP‐3. Together, these target proteins also displayed differential expression patterns in mammalian expression systems. Previous characterization of protein production, showed TIMP‐4 was poorly secreted in CHO cells 26. In contrast, PAI‐1 was detected in the culture medium in high amounts (Fig. S4a and 47) whereas ARTN was not detectable in the culture medium and was present in low amounts in cell extracts (Fig. S4b).

Predicted structures for ARTN, PAI‐1 and TIMP‐4 along with TIMP‐2 and TIMP‐3 were ranked in order of the observed protein production, good (PAI‐1 and TIMP‐2), poor (TIMP‐4) and no detectable production (TIMP‐3 and ARTN). For both hydrophobicity (Fig. S5a) and electrostatic potential (Fig. S5b) analyses, unfavourable surface features (nonpolar and/or positively charged regions) correlated with decreased protein secretion.

Hydrophobicity analysis of TIMP‐4 displayed nonpolar patches present in the C‐terminus common to both TIMP‐2 and TIMP‐3. The surface hydrophobicity of TIMP‐4 was similar to TIMP‐2 and distinct nonpolar patches identified for TIMP‐3 were absent. TIMP‐4 was observed to have a positively charged surface spread over the whole structure in areas that were distinct from and common to both TIMP‐2 and TIMP‐3 (Fig. S5b). A high PosQ value was predicted for TIMP‐4 (3.026), placing it between values gained for TIMP‐2 and TIMP‐3 (Table 1).

ARTN structures have a large hydrophobic patch due to a long stretch of hydrophobic amino acids (S71 to P98) and a large positively charged patch (Fig. S5b). Although the PosQ value for ARTN (1.539, Table 1) was low, the observations from ARTN electrostatic potential analyses were consistent with published reports where ARTN has shown to have a positively charged surface 54.

The well‐secreted target, PAI‐1, has a largely neutral/weakly polar surface (Fig. S5a) and electrostatic potential revealed a largely negatively charged surface (Fig. S5b). This observation correlated with a lower PosQ value predicted for PAI‐1 (1.132) compared to the other targets (Table 1). With the absence of hydrophobic and/or positively charged surface patches associated with poor solubility, one would predict that PAI‐1 would be secreted effectively, which is consistent with previous characterization of PAI‐1 production in CHO cells (Fig. S4a and 47).

Together, comparison of all protein structures showed the presence of large positively charged and/or hydrophobic patches correlated to poor or no detectable protein secretion. The composition of these patches along with the structural position was specific to each protein. Screening of amino acid sequences using this computational approach could potentially identify unfavourable sequence features, which may limit secretion, and rationalize the re‐design of these attributes prior to expression in mammalian cells. Further engineering of these features, such as described for TIMP‐3, could be employed to overcome challenges in the production of other ‘difficult‐to‐express’ recombinants targets for applications where mutation and/or chimeras are acceptable and no change in the functional activity is observed.

## Discussion

Amino acid sequences and secondary structures of specific proteins contain features associated with limitations in recombinant protein production in CHO cells 30, 31, 32. Functionally related proteins TIMP‐2 (well‐secreted) and TIMP‐3 (poorly secreted), with significant sequence and structural identity have been used as models to test sequence‐specific determinants of secretion in a transient CHO system 26. This approach models how a combination of protein engineering strategies and computational analysis defines protein‐specific or protein generic structural determinants assessed in other model proteins (ARTN, PAI‐1 and TIMP‐4).

Domain swapping experiments with TIMP‐2 and TIMP‐3 identified regions that contribute to restrictions in protein secretion. Mature NT2/CT3, containing a single N‐glycan site, was produced in amounts comparable to TIMP‐2. In contrast, NT3/CT2 was not detectable in culture medium but was present intracellularly. Glycosidase treatment of NT2/CT3 in the culture medium proved the presence of complex‐type, fully processed (Endo H‐insensitive) glycans, as well as the presence of Endo H‐sensitive, high mannose/hybrid forms. These high mannose/hybrid forms in the culture medium may result as a consequence of the high flux of protein going through the ER and Golgi causing some glycoforms to escape final processing. Alternatively, high mannose/hybrids forms may be released early as a result of cell lysis. The detection of high mannose/hybrid glycoforms has been reported for other secreted recombinant proteins in mammalian cells 55, 56, 57, 58.

Domain swapping suggested sequences within the N‐terminal domain of TIMP‐3 restricted successful secretion. The same approach could be applied to exchange domains between TIMP‐2 (well‐secreted) and TIMP‐4 (poorly secreted) due to the high sequence similarity, to identify limiting unfavourable regions. Alternatively, whether addition of the TIMP‐3 N‐terminus acts to limit secretion of any model protein. In cases where there is a lack of structural homology, for example with ARTN (unsuccessfully secreted), mutants could be generated to assess the effect of amino acid sequence modifications on protein production. To further define processes that may be limiting, fluorescent tags could be used to gain further understanding of protein folding and secretion using real‐time microscopy techniques.

The algorithms used to map surface properties (electrostatic potential and hydrophobicity) are based on a protein solubility database of *E. coli* proteins 51. This study has shown that the algorithm can be implemented in the context of recombinant protein production in a mammalian expression system. Use of the algorithm in combination with protein engineering efforts led to the design of a modified TIMP‐3 construct (enTIMP‐3), which was successfully secreted in a fully processed form. However, although enTIMP‐3 was detected in a secreted form, the activity was not measured. The purpose of this study was to examine effects on protein secretion, however, further studies could determine accurate protein titres and whether a change in the functional activity of this chimera is observed.

Analysis of different recombinant target protein structures using computational tools 37, 46 showed an increase in unfavourable surface features, such as positively charged and/or nonpolar regions, correlated with decreasing protein production. Although the types of features were broadly the same for poorly secreted exemplars, unsurprisingly perhaps, the specific nature of the features was determined by the unique amino acid composition of the proteins. For example, TIMP‐3 sequences contained a greater proportion of lysine residues, whereas ARTN contained more arginine residues. In certain cases, some proteins displayed unfavourable characteristics from both electrostatic and hydrophobicity analyses. TIMP‐3 and ARTN displayed positively charged regions on their surface as well as exposed nonpolar regions. Therefore, it is possible that either surface charge or hydrophobicity act independently or in combination to prevent efficient protein overproduction. The association between certain unfavourable sequence/structural features and the cellular processes that subsequently act to limit production of these target proteins was not within the scope of this study but presents future avenues for exploration.

For electrostatic potential analysis, a large maximum positive potential patch size (PosQ value) correlated with decreased protein production. For example, poorly secreted TIMP‐4 contained a large positively charged surface which may contribute to limitations in its post‐translational processing and poor secretion. Further interrogation of this computational method with a wider panel of recombinant targets could provide an effective tool to report on the predictability of mammalian protein production. Screening of protein structures using the described computational approach, could act to identify unfavourable sequence features and aid redesign of ‘difficult’ recombinant targets prior to expression studies and aid efficient protein production.

## Author contributions

AJD, WMA, DIF and RGR conceived and/or supervised the study. HH designed and performed the experiments. Computational analysis was completed in collaboration with JW and MAC. HH drafted the manuscript and AJD, DIF and JW made manuscript revisions.

## Supporting information


**Fig. S1.** Western blot analysis of TIMP domain‐exchanged sequences in transfected CHO cell culture samples with specific primary antibodies.
**Fig. S2.** Glycosidase treatment of intracellular and secreted NT2/CT3 and enTIMP‐3 protein.
**Fig. S3.** Computational analyses of TIMP domain exchanged structures.
**Fig. S4.** Transient expression of murine Plasminogen activator inhibitor (PAI‐1) and Artemin (ARTN) sequences in CHO cell cultures.
**Fig. S5.** Comparison of the surface properties for all protein structures.
