# Supplementary material for: A protein chimera strategy supports production of a model “difficult‐to‐express” recombinant target
Source: FEBS Lett. 2018 Jul 3;592(14):2499–511. doi: 10.1002/1873-3468.13170 (PMC6174982; doi:10.1002/1873-3468.13170)

(a) Transient expression of murine Plasminogen activator inhibitor (PAI-1)

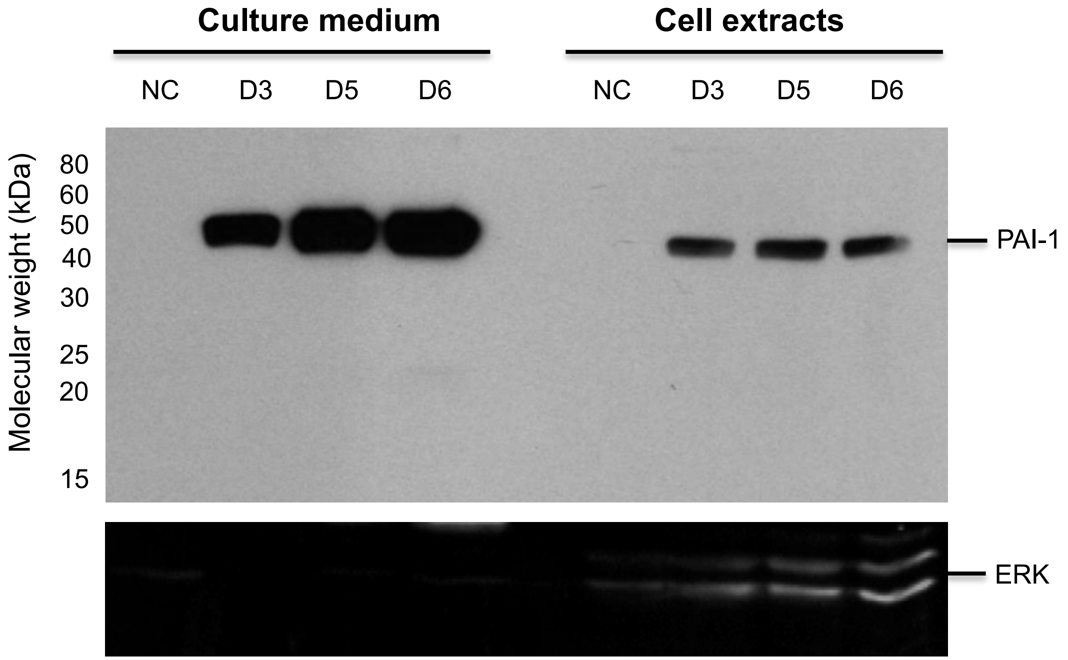

(b) Transient expression of Artemin (ARTN)

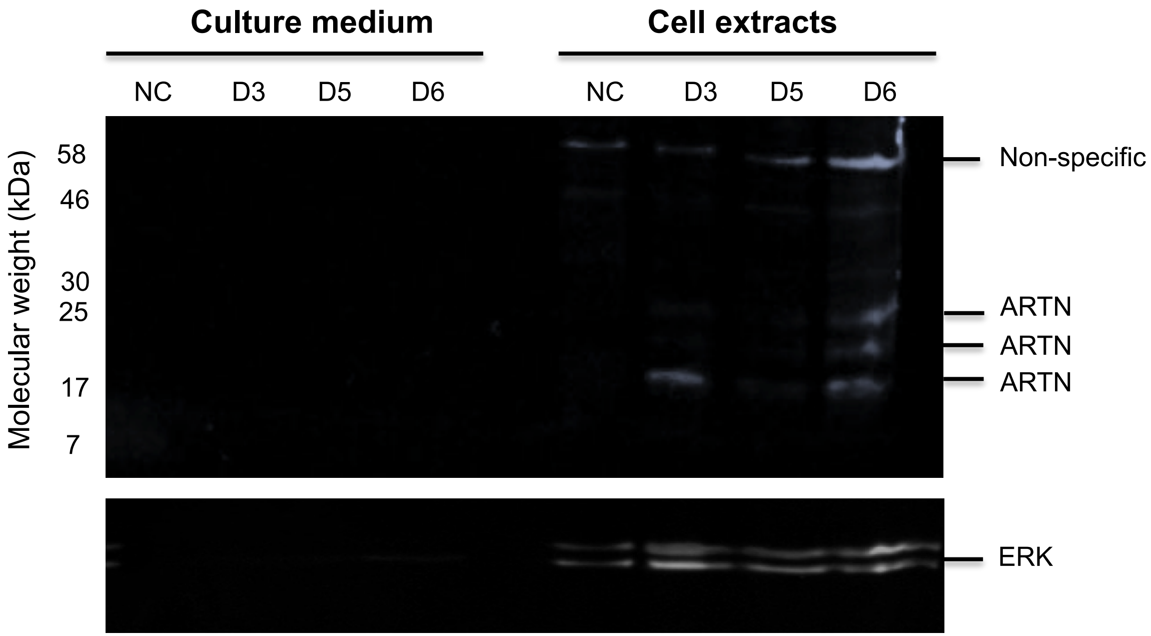

Supplement: Supplementary file 1 — Fig. S1. Western blot analysis of TIMP domain‐exchanged sequences in transfected CHO cell culture samples with specific primary antibodies. Fig. S2. Glycosidase treatment of intracellular and secreted NT2/CT3 and enTIMP‐3 protein. Fig. S3. Computational analyses of TIMP domain exchanged structures. Fig. S4. Transient expression of murine Plasminogen activator inhibitor (PAI‐1) and Artemin (ARTN) sequences in CHO cell cultures. Fig. S5. Comparison of the surface properties for all protein structures. [file FEB2-592-2499-s001.zip › Figures - Hirra Hussain 16.pdf]
