# Supplementary figures and images for: A protein chimera strategy supports production of a model “difficult‐to‐express” recombinant target
Source: FEBS Lett. 2018 Jul 3;592(14):2499–511. doi: 10.1002/1873-3468.13170 (PMC6174982; doi:10.1002/1873-3468.13170)

**(b) Detection using TIMP-3 specific antibody**

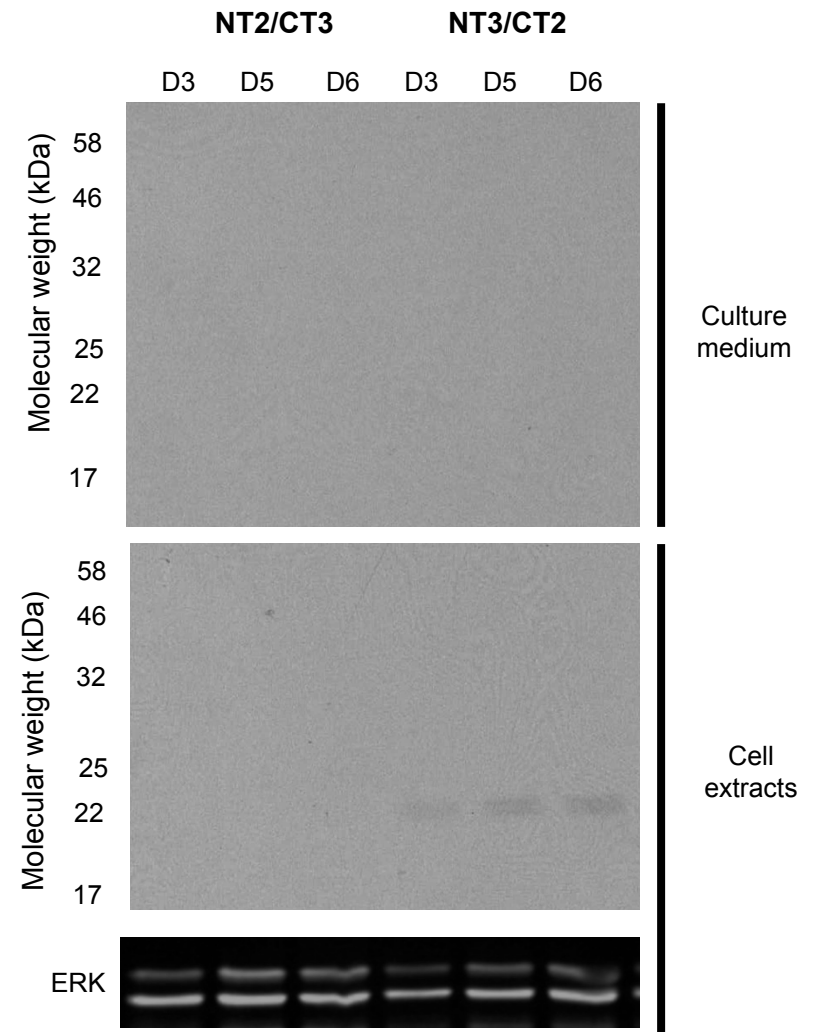

Supplement: Supplementary file 1 — Fig. S1. Western blot analysis of TIMP domain‐exchanged sequences in transfected CHO cell culture samples with specific primary antibodies. Fig. S2. Glycosidase treatment of intracellular and secreted NT2/CT3 and enTIMP‐3 protein. Fig. S3. Computational analyses of TIMP domain exchanged structures. Fig. S4. Transient expression of murine Plasminogen activator inhibitor (PAI‐1) and Artemin (ARTN) sequences in CHO cell cultures. Fig. S5. Comparison of the surface properties for all protein structures. [file FEB2-592-2499-s001.zip › Figures - Hirra Hussain 11.pdf]

(a) Glycosidase treatment of NT2/CT3

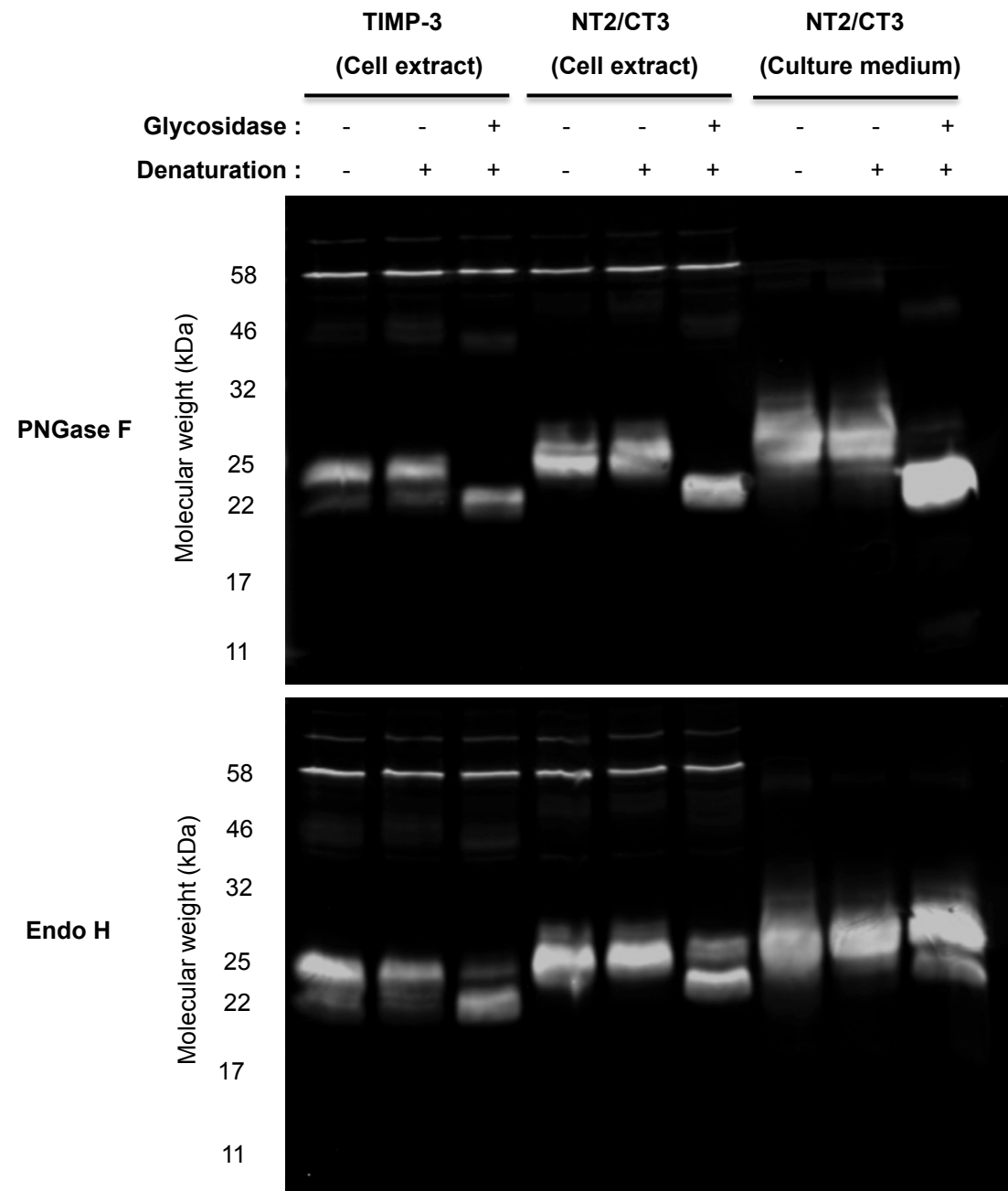

Supplement: Supplementary file 1 — Fig. S1. Western blot analysis of TIMP domain‐exchanged sequences in transfected CHO cell culture samples with specific primary antibodies. Fig. S2. Glycosidase treatment of intracellular and secreted NT2/CT3 and enTIMP‐3 protein. Fig. S3. Computational analyses of TIMP domain exchanged structures. Fig. S4. Transient expression of murine Plasminogen activator inhibitor (PAI‐1) and Artemin (ARTN) sequences in CHO cell cultures. Fig. S5. Comparison of the surface properties for all protein structures. [file FEB2-592-2499-s001.zip › Figures - Hirra Hussain 12.pdf]

(b) Glycosidase treatment of engineered TIMP-3 (enTIMP-3)

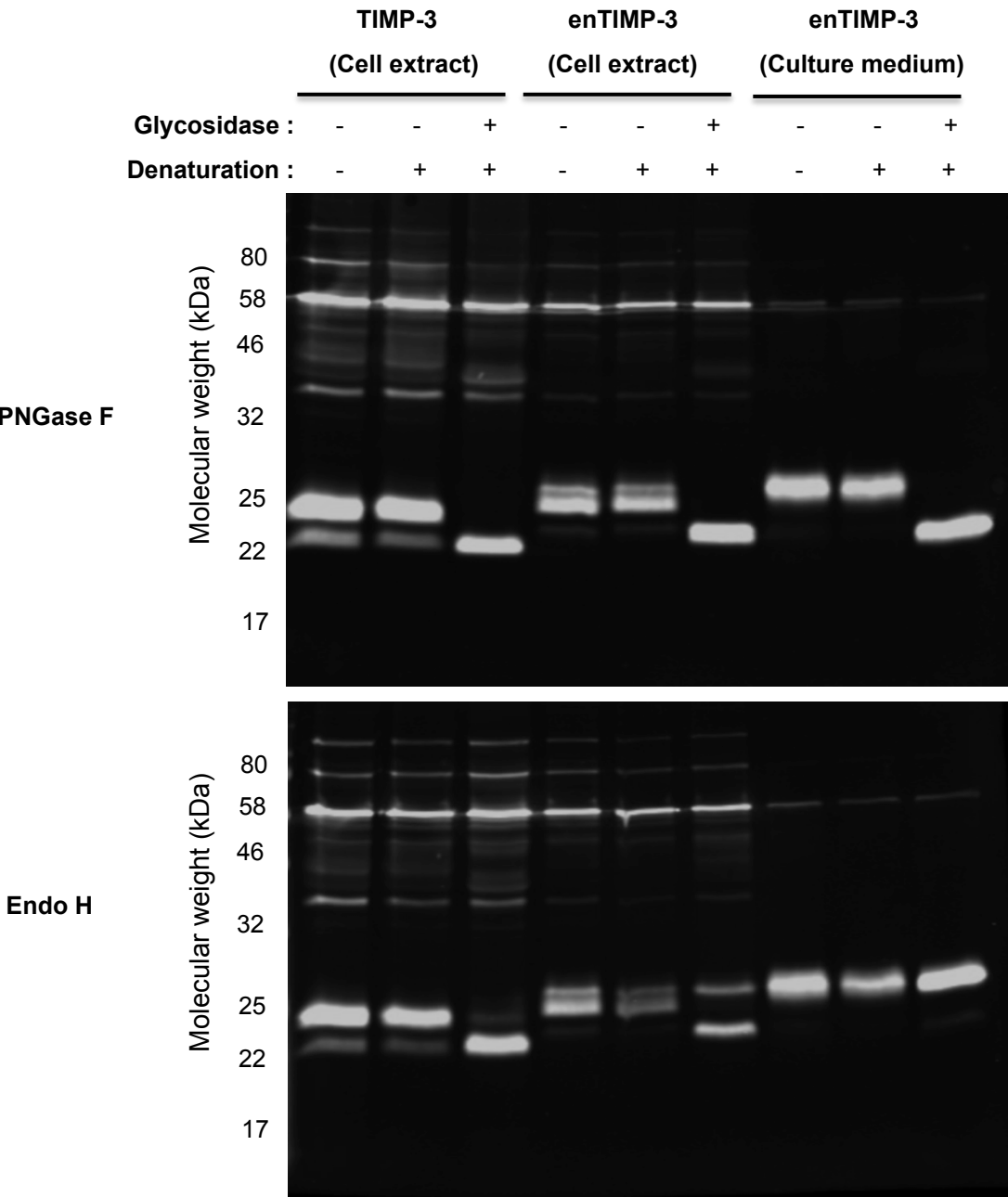

Supplement: Supplementary file 1 — Fig. S1. Western blot analysis of TIMP domain‐exchanged sequences in transfected CHO cell culture samples with specific primary antibodies. Fig. S2. Glycosidase treatment of intracellular and secreted NT2/CT3 and enTIMP‐3 protein. Fig. S3. Computational analyses of TIMP domain exchanged structures. Fig. S4. Transient expression of murine Plasminogen activator inhibitor (PAI‐1) and Artemin (ARTN) sequences in CHO cell cultures. Fig. S5. Comparison of the surface properties for all protein structures. [file FEB2-592-2499-s001.zip › Figures - Hirra Hussain 13.pdf]

(a) Hydrophobicity analysis of domain-exchanged structures

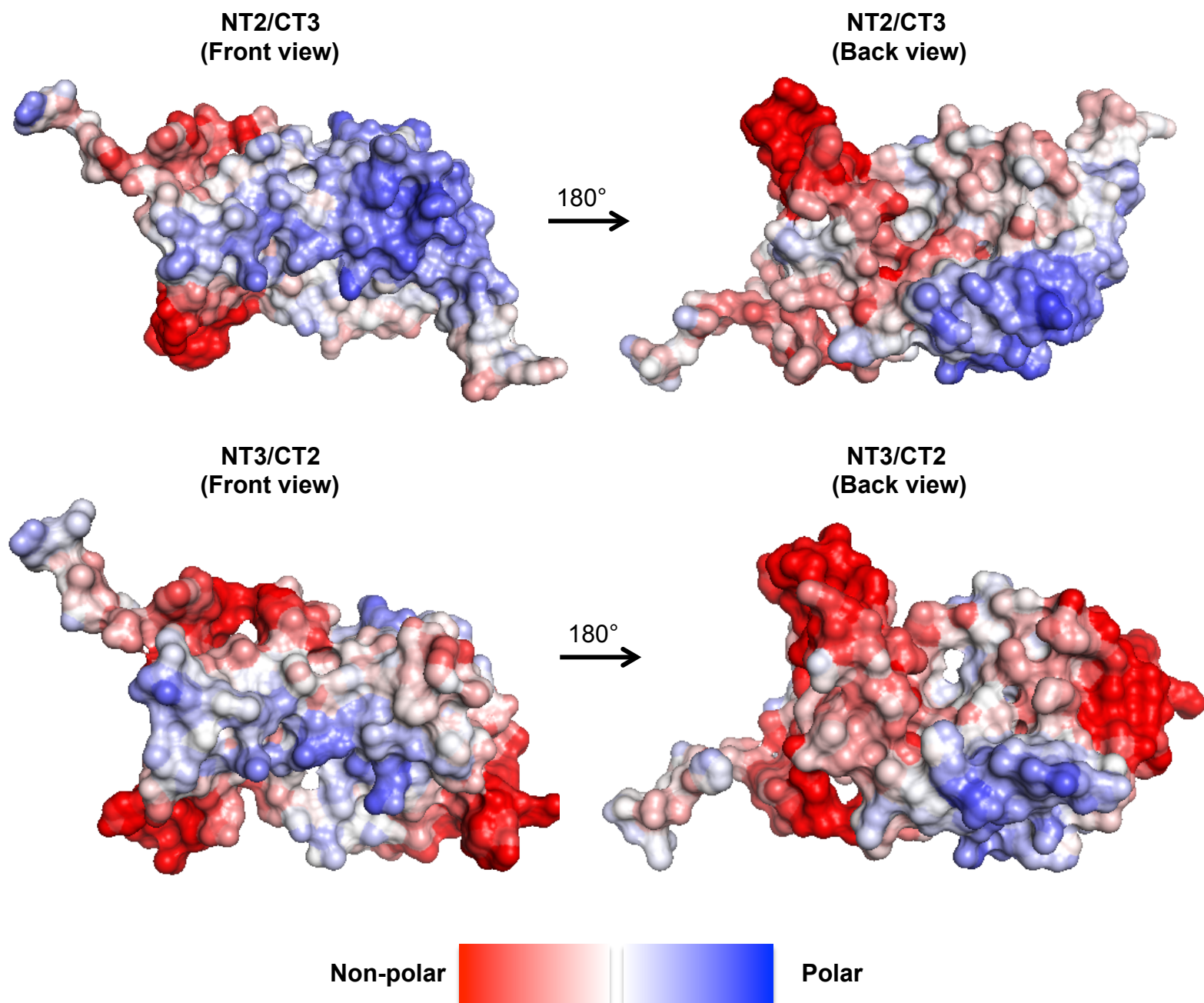

Supplement: Supplementary file 1 — Fig. S1. Western blot analysis of TIMP domain‐exchanged sequences in transfected CHO cell culture samples with specific primary antibodies. Fig. S2. Glycosidase treatment of intracellular and secreted NT2/CT3 and enTIMP‐3 protein. Fig. S3. Computational analyses of TIMP domain exchanged structures. Fig. S4. Transient expression of murine Plasminogen activator inhibitor (PAI‐1) and Artemin (ARTN) sequences in CHO cell cultures. Fig. S5. Comparison of the surface properties for all protein structures. [file FEB2-592-2499-s001.zip › Figures - Hirra Hussain 14.pdf]

(b) Electrostatic potential analysis of domain-exchanged structures

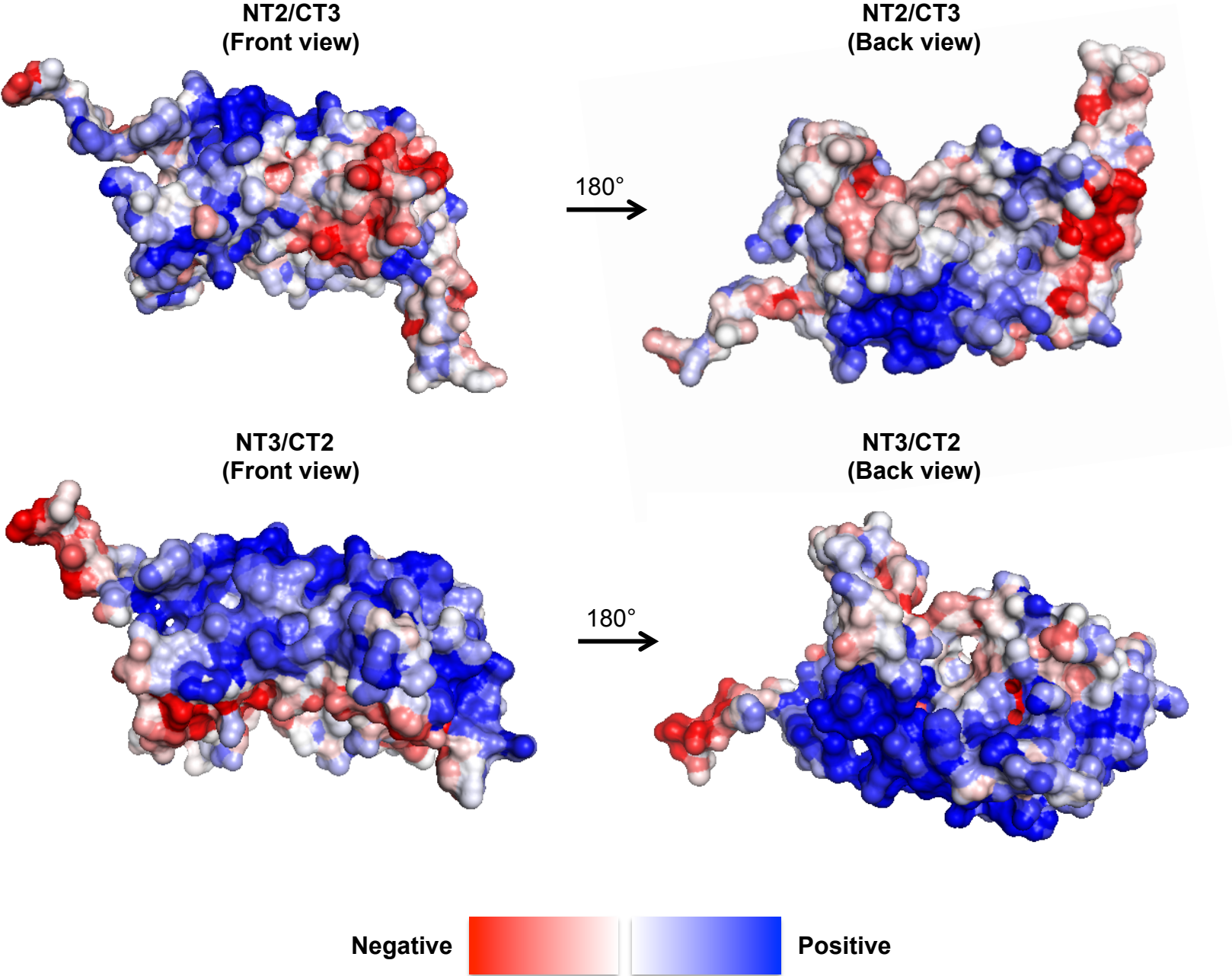

Supplement: Supplementary file 1 — Fig. S1. Western blot analysis of TIMP domain‐exchanged sequences in transfected CHO cell culture samples with specific primary antibodies. Fig. S2. Glycosidase treatment of intracellular and secreted NT2/CT3 and enTIMP‐3 protein. Fig. S3. Computational analyses of TIMP domain exchanged structures. Fig. S4. Transient expression of murine Plasminogen activator inhibitor (PAI‐1) and Artemin (ARTN) sequences in CHO cell cultures. Fig. S5. Comparison of the surface properties for all protein structures. [file FEB2-592-2499-s001.zip › Figures - Hirra Hussain 15.pdf]
