# Supplementary material for: A protein chimera strategy supports production of a model “difficult‐to‐express” recombinant target
Source: FEBS Lett. 2018 Jul 3;592(14):2499–511. doi: 10.1002/1873-3468.13170 (PMC6174982; doi:10.1002/1873-3468.13170)
Supplement: Supplementary file 2 [file FEB2-592-2499-s002.docx]

# List of Figures in Supplementary Material

Supplementary Figure S1 Western blot analysis of TIMP domain-exchanged sequences in transfected CHO cell culture samples with specific primary antibodies.

Transfected cultures were sampled for secreted and intracellular protein on days 3, 5 and 6 (D3. 5 and 6 respectively) and prepared under reducing conditions. Samples were analysed by western blot using **a**) a polyclonal anti-TIMP2 primary antibody and (**b**) polyclonal anti-TIMP3 primary antibody for detection. The polyclonal anti-TIMP-3 antibody targets loop 1 in the N-terminus of TIMP-3. Data shown is representative of two biological replicates.

Supplementary Figure S2 Glycosidase treatment of intracellular and secreted NT2/CT3 and enTIMP-3 protein.

Cell lysates and culture supernatant from **a**) NT2/CT3-transfected and **b**) enTIMP-3-transfected cultures along with cell extracts from TIMP-3 cultures (all sampled on day 5 post-transfection) were treated with N-glycosidase F (PNGase F) and Endoglycosidase H (Endo H) separately. Untreated, denatured, and treated samples were analysed by western blot. Data is representative of three biological replicates.

Supplementary Figure S3 Computational analyses of TIMP domain exchanged structures.

Structural models of NT2/CT3 and NT3/CT2 were analysed for surface **a**) hydrophobicity and **b**) electrostatic potential. In each case, surface map views of the front and back (180° rotation) are shown.

Supplementary Figure S4 Transient expression of murine Plasminogen activator inhibitor (PAI-1) and Artemin (ARTN) sequences in CHO cell cultures.

CHO-EBNA-GS cells were transiently transfected and sampled for secreted and intracellular proteins on day 3, day 5 and day 6 (D3, D5 and D6) post-transfection. Protein samples for **a**) PAI-1 and **b**) ARTN were analysed by western blot. Non-transfected cells (NC) were loaded as controls. ERK was used as a loading control for cell extracts. Data is representative of at least four biological replicates.

Supplementary Figure S5 Comparison of the surface properties for all protein structures.

Predicted surface **a)** hydrophobicity (*top panel*, front view) and **b)** electrostatic potential (*bottom panel*, back view) maps are shown for all protein structures mPAI-1, TIMP-2, enTIMP-3, TIMP-4, TIMP-3 and ARTN. Structures are shown in order of the observed protein secretion: good (mPAI-1, TIMP-2, enTIMP-3), poor (TIMP-4) and no detectable secretion (TIMP-3, ARTN). To visualise the main patches of interest, the front view is shown for each structure for hydrophobicity analysis and back view for electrostatic potential analysis.
